# Lentiviral vector‐encoded microRNA‐based shRNA‐mediated gene knockdown of *N*‐methyl‐D‐aspartate receptors in skin reduces pain

**DOI:** 10.1002/brb3.587

**Published:** 2016-11-04

**Authors:** Chien‐Cheng Liu, Jiin‐Tsuey Cheng, Kuo‐Chuan Hung, Yuan‐Yi Chia, Ping‐Heng Tan

**Affiliations:** ^1^ Department of AnesthesiologyE‐DA HospitalKaohsiungTaiwan; ^2^ Department of Biological SciencesNational Sun Yat‐Sen UniversityKaohsiungTaiwan; ^3^ Department of AnesthesiologyKaohsiung Veterans General HospitalKaohsiungTaiwan; ^4^ School of MedicineI‐Shou UniversityKaohsiungTaiwan

**Keywords:** analgesia, intradermal injection, NMDA receptor, shRNAmir

## Abstract

**Background and Purpose:**

RNA polymerase II promoters that drive the expression of rationally designed primary microRNA‐based shRNA, for example, shRNAmir, can produce more potent gene knockdown than RNA polymerase III promoters. Antagonists of peripheral *N* methyl‐D‐aspartate (NMDA) receptors that do not interfere with central glutamate processing would prevent the development of adverse central nervous system effects. Thus, in this study, we examined the effects of gene silencing and antinociception on formalin‐ and Complete Freund's adjuvant (CFA)‐induced pain in rats by subcutaneously injecting a lentiviral vector encoding a shRNAmir that targets the NR1 subunit of the NMDA receptor.

**Methods:**

Rats received intradermal injections of different doses of NR1 shRNAmir at different time points before injection of formalin. Pain behavior was assessed by monitoring the paw flinch response, paw withdrawal threshold, and thermal withdrawal latency. We then analyzed NR1 messenger RNA and protein expression in skin and the L5 dorsal root ganglion (DRG).

**Results:**

We found that intradermal injection of 1, 5, and 10 μg of shRNAmir significantly inhibited flinch responses (*p* < .05). Administration of 5 μg of shRNAmir resulted in the attenuation of CFA‐induced mechanical allodynia, but did not affect the time spent on the rotarod. Real‐time polymerase chain reaction and western blotting revealed that NR1 mRNA and protein levels were significantly lower in all NR1 shRNAmir1 groups than in controls (*p* < .05). There was a significant reduction in the percentage of NR1‐ and pERK‐positive neurons in the DRG ipsilateral to shRNAmir treated paws (*p* < .05). The effect of antinociception and inhibition of NR1 expression by NR1 shRNAmir was evident on day 3 and persisted for 7 days after injection of 5 μg of vector.

**Conclusion:**

Peripheral administration of the vector‐encoded NR1 shRNAmir is a promising therapy for persistent inflammatory pain.

## 1 Introduction

MicroRNAs (miRNA) are transcribed as long primary transcripts by the RNase III enzyme, Drosha, and then processed into mature miRNAs in the cytoplasm (Cullen, 2004; Elbashir, Lendeckel, & Tuschl, 2001; Lee et al., 2003). Cullen and colleagues produced a template of miRNA‐30 precursor RNA, in which miRNA‐30 was replaced by a target gene sequence and found that it effectively inhibited the expression of the target gene (Zeng, Cai, & Cullen, 2005). Furthermore, miRNA‐30‐based short hairpin RNAs (shRNAs), also called shRNAmir, have been shown to inhibit gene expression 12 times more potently than traditional stem‐loop shRNAs (Silva et al., 2005). To achieve long‐term gene silencing, shRNAs expressed from plasmids or viral vectors can be used instead of small interfering RNAs (siRNAs).

It is well known that certain *N*‐methyl‐D‐aspartate (NMDA) receptor antagonists are useful analgesics for managing inflammatory (Beirith, Santos, & Calixto, 2002; Davidson, Coggeshall, & Carlton, 1997) and burning pain in humans (Warncke, Jorum, & Stubhaug, 1997). Nonetheless, the use of these antagonists is limited because of the numerous side effects resulting from antagonizing NMDA receptors in the central nervous system (CNS). Thus, inhibition of peripheral NMDA receptors is an alternative analgesic option. Intrathecal delivery of synthetic siRNAs to the spinal cord has successfully achieved silencing of molecular targets in various models of neurological disease including pain (Dorn et al., 2004; Garraway, Xu, & Inturrisi, 2009; Luo et al., 2005). In our previous study (Tan et al., 2010), we demonstrated that local peripheral injection of NR1 siRNA induced effective and specific silencing of the NMDA receptor and that it significantly attenuated formalin‐induced nociceptive behaviors without producing significant side effects. However, synthetic siRNA‐mediated RNAi has a transient effect, with cells recovering after a single treatment. We hypothesized that vector‐encoded shRNAmir‐mediated gene knockdown of NMDA receptors in subcutaneous tissues may provide a longer period of analgesia. To test this hypothesis, we examined the gene silencing and antinociception effects of intradermal injections of a shRNAmir that targets the NR1 subunit of the NMDA receptor in rat models of formalin‐ and Complete Freund's adjuvant (CFA)‐induced nociception.

## 2 Methods

### 2.1 Generation of lentiviral vector expressing shRNAmir

The sequences for the shRNAmirs for *NR1* were designed based on GenBank accession number NM 017010. The following sequences were the target sequences tested: sequence 1, GGGTAAACAACAGCAACAA (shRNAmir1); sequence 2, AGACTAAAGATAGTGACAA (shRNAmir2); sequence 3, nonsilencing shRNAmir (NS‐shRNAmir) (Nonsilencing lentiviral shRNAmir, RHS 4346, Open Biosystems, Huntsville, AL, USA). No significant homology to known rat gene sequences was found in the GenBank database.

Lentiviral vectors encoding shRNAmirs were made using the backbone of the pGIPZ vector (Open Biosystems), which was remodeled to express green fluorescent protein and shRNAmirs from a bicistronic transcript driven by a cytomegalovirus (CMV) promoter, followed by a PURO resistance gene (pGIPZ‐shRNAmir). The template “miRNA30‐like” DNA oligonucleotides targeting two positions of NR1 mRNAs (shRNAmir1 and shRNAmir2) were ligated into the *Xho*I/*Mlu*I sites in the pGIPZ‐shRNAmir vector (Open Biosystems). An additional control vector (pGIPZ nonsilencing shRNAmir lentiviral control vector, RHS 4346) was obtained from Open Biosystems. The nonsilencing shRNAmir sequence containing no homology to known mammalian genes was expressed under the control of the CMV promoter. The shRNAmirs were given at a ratio of 1 μl of 100 mmol/L polyethyleneimine (PEI) (Glen Burnie, MD, USA) solution per 5 μg of shRNAmir. The shRNAmir‐PEI complexes and PEI alone were diluted to a total volume of 100 μl with 5% dextrose in water and mixed for 10 min at room temperature before injection.

### 2.2 Intradermal injection of vector expressing shRNAmir

A total of 139 male Sprague–Dawley rats, weighing 250–350 g, were used in this study and all procedures adhered to the guidelines for animal pain research (Zimmermann, 1983). All animal protocols were approved by the Institutional Review Board of I‐Shou University, Kaohsiung, Taiwan. Two different sequences of shRNAmir, namely shRNAmir1 and shRNAmir2 (5 μg each, *n* = 6 rats in each group), were tested for their antinociceptive and gene silencing effects. The controls received local injection of 1 μl PEI (vehicle group, *n* = 6) or 100 μl saline (saline group, *n* = 6). To examine the effects of different doses, we administered three doses of NR1 shRNAmir1 namely 1, 5, and 10 μg (*n* = 6 rats in each group). Controls received intradermal injections of 100 μl saline, 1 μl PEI, or 5 μg NR1 NS‐shRNAmir (*n* = 6 in each group). All injections were performed 3 days prior to formalin injection. The rats were further evaluated by the rotarod test before the administration of formalin in the control groups and in the group that received 5 μg NR1‐shRNAmir1. To exclude the systemic analgesic effect of NR1 shRNAmir1, rats (*n* = 6) received local injection of 5 μg NR1 shRNAmir1 in one paw followed by injection of formalin in the contralateral paw 3 days later. Skin tissue was taken immediately after the behavioral tests for analysis of NR1 mRNA by real‐time polymerase chain reaction (PCR) and NR1 protein by western blotting. The duration of the NR1 shRNAmir effect was tested on three groups of rats that received 5 μg NR1 shRNAmir1 1, 3, 7, and 14 days before formalin testing (*n* = 6 in each group). Rats that received 1 μl PEI at the same time points before formalin testing served as the control groups (*n* = 6 in each time period group). Skin tissues were dissected immediately after behavioral testing in each group for analysis of NR1 by real‐time PCR and western blotting. In the groups of rats that received NR1 shRNAmir1 or PEI 3 days before formalin testing, skin tissue was also analyzed for NR 2A, B, C, D by real‐time PCR. To test for the expression of NR1 in the dorsal root ganglion (DRG), the left and right L5 DRGs were dissected for immunohistochemical staining of NR1 3 days after injection of 5 μg of NR1 shRNAmir1 in the left paw in four rats. To examine the antinociceptive effect of NR1 shRNAmir on CFA‐induced mechanical allodynia, rats received 5 μg of NR1 shRNAmir1 in the left paw. Controls received intradermal injections of 100 μl saline or 1 μl PEI (*n* = 5 in each group). All injections were performed 2 days prior to CFA injection. Mechanical withdrawal threshold was tested 1 day after CFA injection. After behavioral testing, the left L5 DRGs were dissected from rats in the saline and shRNAmir groups for immunohistochemical staining of phosphor‐extracellular signal‐regulated kinase 1 and 2 (pERK1/2). Rats received 5 μg of NR1 shRNAmir1 in the left paw to examine the behavioral effects and the changes in expression levels of NMDA receptor subunits and interferon‐α in the DRG after intradermal injection of NR1 shRNAmir. Controls received intradermal injections of 100 μl saline or 1 μl PEI (*n* = 4 in each group). Mechanical withdrawal threshold and thermal hyperalgesia were assayed 3 days after NR1 shRNAmir injection. The bilateral L4‐6 DRGs in rats that received injection of NR1 shRNAmir were dissected after behavioral testing and then analyzed by real‐time PCR for the expression of NR1, NR2A, B, C, D, and interferon‐α.

### 2.3 Behavioral tests

#### 2.3.1 Formalin assay

Before formalin injection, the rats were placed inside the testing chamber for at least 30 min to acclimate. The rats then received an injection of 50 μl 1% formalin solution into the paw to evoke paw flinching behavior that persists for approximately 1 hr. Following injection, the rats were immediately returned to their testing chamber. The number of paw flinches was counted every min during the first 5 min, and then for 1 min per 5 min until 60 min following injection of formalin in each rat.

#### 2.3.2 CFA assay

Complete Freund's adjuvant (0.1 ml; Sigma, St. Louis, MO, USA) was subcutaneously injected into the plantar hind paw to induce inflammation. To test for mechanical allodynia, animals were put into a plastic box (11 × 13 × 24 cm) on an elevated metal mesh floor and allowed 30 min for habituation. Mechanical paw withdrawal thresholds (PWTs) were determined using the methods described by Chaplan, Bach, Pogrel, Chung and Yaksh (1994). Briefly, the hind paw was pressed with one of a series of von Frey hairs with logarithmically incremental stiffness (0.6, 1, 1.4, 2, 4, 6, 8, 10, 15, and 26 g; Stoelting, Wood Dale, IL, USA) presented perpendicular to the plantar surface for 4–5 s for each hair. The 50% withdrawal threshold was determined using the up–down method by Dixon (1980). The animals were tested 1 day before injection of treatment agent for baseline data and then 1 day after injection of CFA.

#### 2.3.3 Rotarod test

Rats were trained for 2 days before the test to remain for 30 s on an accelerating Ugo Basile rotarod apparatus at 12 rpm. The rats were then placed on a rotarod set at 40 rpm. The length of time (in seconds) that the rat remained on the rod was measured. A cutoff of 60 s was observed (Boyce et al., 1999).

### 2.4 Quantitative real‐time PCR

Real‐time PCR was performed using the ABI Prism 7500 Sequence Detection System (Applied Biosystems, Foster City, CA, USA) with SYBR Green detection. The following PCR program was used: stage 1, 50°C for 3 min; stage 2, 95°C for 10 min; stage 3, 40 cycles of 15 s at 95°C and 45 s at 60°C. The program ended at 25°C. β‐actin was chosen as the endogenous control in the analysis of skin and glyceraldehyde‐3‐phosphate dehydrogenase was used as the control in the analysis of DRG. To exclude whether observed effects were due to induction of an interferon response, we also analyzed the mRNA level of interferon‐α. mRNA levels of NR1 and NR2 subunits and interferon‐α were analyzed. Primer sequences were designed by Primer Express^®^ Software v3.0.1 (Thermo Fisher Scientific Inc., USA) and analyzed for specificity using the nucleotide Basic Local Alignment Search Tool and Primer‐BLAST (http://blast.ncbi.nlm.nih.gov/Blast.cgi). The PCR primer sequences for NR1 and NR2 subunits of the NMDA receptor and for interferon‐α are listed in Table 1. For each PCR reaction, 12.5 μl of 2× SYBR Green PCR Master Mix (ABI, Foster City, CA) and 1.0 μl (10 μmol) of the desired primer mixture were added to the complementary DNA templates to reach a final volume of 25 μl. The PCR setup was singleplex, that is, the target and reference genes were detected in separate tubes. A “no‐template” control consisting of water (sterile and ultraviolet irradiated) was used for each primer pair.

**Table 1:** Real‐time polymerase chain reaction primers

Gene	Direction	Primers
*NR1*	Fwd.	5′‐GCG ACT CCC GCA GCA AT‐3′
	Rev.	5′‐CCC CTG CCA TGT TCT CAA AA‐3′
*NR2A*	Fwd.	5′‐TCC ACT CAA GGA ATC TTG TGA GAT AT‐3′
	Rev.	5′‐ACT TGC CCA TGT GTA TTT ATT TGT TT‐3′
*NR2B*	Fwd.	5′‐AAC CCT CGT GGC CAG CA‐3′
	Rev.	5′‐GGT GGA CAG ATG CGG GAA‐3′
*NR2C*	Fwd.	5′‐GGC CCA GCT TTT GAC CTT AGT‐3′
	Rev.	5′‐CCT GTG ACC ACC GCA AGA G‐3′
*NR2D*	Fwd.	5′‐AGG GTT TCT GCA TTG ATA TTC TGA A‐3′
	Rev.	5′‐TCA CCA ATC ATG CCA TTC CA‐3′
*Interferon‐α*	Fwd.	5′‐GGG ATG CAA CCC TCC TAG ACT‐3′
	Rev.	5′‐ACA GGC TTG CAG ACC ACT CA‐3′
*β‐actin*	Fwd.	5′‐CGT ACC ACT GGC ATT GTG ATG‐3′
	Rev.	5′‐CAC GCT CGG TCA GGA TCT TC‐3′
*GAPDH*	Fwd.	5′‐TGC CCC CAT GTT TGT GAT G‐3′
	Rev.	5′‐GCT GAC AAT CTT GAG GGA GTT GT‐3′

The NR1, NR2A‐D subunit, interferon‐α, β‐actin, and glyceraldehyde‐3‐phosphate dehydrogenase (GAPDH) primer sequences were derived from the National Center for Biotechnology Information (Bethesda, Virginia) nucleotide sequence (accession numbers U11418, AF001423, NM_012574, NM_012575, NM_022797, NM_001106667, NM_031144, and NM_017008, respectively).

Each target gene amplification was analyzed and the melting curve analysis indicating the temperature of dissociation showed a single peak. The results of the PCR analysis were analyzed by calculating threshold cycle (*C*_t_) values of target gene mRNA and endogenous gene mRNA. Differences between the *C*_t_ values of the target gene NR1, each NR2 subunit, interferon‐α, and the endogenous control gene were calculated and are shown as ∆*C*_t_. Subsequently, differences between the ∆*C*_t_ in the treated groups and the ∆*C*_t_ in the control group in skin tissue and the ∆*C*_t_ of the right DRGs were calculated as ∆∆*C*_t_. The relative expression of target mRNA present in the skin and DRG in treated groups versus that in the control groups are shown as $2^{-\Delta \Delta C_{t}}$.

### 2.5 Western blots

Protein samples were prepared from spinal cord tissues as previously described (Rabben, Skjelbred, & Oye, 1999) and 30 μg from each sample were separated on 10% SDS‐PAGE gel. After the transfer, the blots were incubated overnight at 4°C with NR1 antibody (rabbit, 1:2,000; Sigma Chemical Company, St. Louis, MO, USA) and actin antibody (mouse, 1:15,000; Millipore, Billerica, MA, USA). For the loading control, the blots were probed with β‐actin antibody. The blots were scanned and analyzed with Image‐Pro Plus analysis software (Media Cybernetics, Silver Spring, MD, USA). The ratio of NR1 immunoreactivity to β‐actin immunoreactivity was calculated.

### 2.6 Immunohistochemistry of DRG

The rats were perfused transcardially with 0.1 mol/L pH 7.4 phosphate‐buffered saline (PBS) followed by 4% paraformaldehyde in PBS. The DRGs were then dissected for immunohistochemical staining. For immunofluorescence analysis, DRG sections were fixed in 4% paraformaldehyde for 15 min and then blocked in Image‐iT Fix Signal Enhancer (Molecular Probes, Carlsbad, CA, USA) for 1 hr at room temperature. The sections were incubated in mouse polyclonal antiglutamate receptor NR1 (1:400; Pharmingen, Franklin Lakes, NJ, USA) or rabbit anti‐pERK1/2 (1:200; Cell Signaling Technology, Danvers, MA, USA) at 4°C overnight. Following overnight incubation, the sections were incubated for 1.5 hr at room temperature in Dylight 488‐conjugated goat anti‐mouse antibody (1:1,000 dilution; Jackson ImmunoResearch Laboratories, West Grove, PA, USA) or FITC‐conjugated goat anti‐rabbit IgG (1:200; Chemicon International, USA). A Nikon Eclipse E800 fluorescence microscope (Nikon Instech Company, Kawasaki, Japan) coupled to a cool digital camera (Diagnostic Instruments Inc., Sterling Heights, MI, USA) and an image‐analyzing system (Advanced Spot Software; Diagnostic Instruments, Inc.) was used to analyze the image.

### 2.7 Image analysis

The images were analyzed by an observer blinded to group assignments. For calculation of immunofluorescence of NR1‐positive neurons in DRG specimens, three DRG sections were selected from the right and left L5 DRG in four rats from each group. For calculation of pERK‐positive neurons in DRG specimens, three DRG sections were selected from the left L5 DRG in five rats from each group. Neurons in which fluorescence intensity was three times higher than background fluorescence were defined as NR1‐ or pERK‐positive neurons. The ratio of NR1‐ and pERK‐positive neurons to total number of neurons was calculated. The immunostaining intensity of each image was analyzed using Image‐Pro Plus analysis software (Media Cybernetics). The signals were analyzed under 200× magnification.

### 2.8 Statistical analysis

All data are expressed as means ± standard deviations (*SD*s). Initially, two‐way analysis of variance (ANOVA) was used to analyze the effects of the different time points and treatments as well as interaction of treatment × control effect. Then, one‐way ANOVA was used to compare flinching number differences at each time point in each group. All analyses were followed by the Student–Newman–Keuls test as part of the multiple comparison analysis. Student's *t* tests were used to compare the data in the time course study in the NR1 mRNA and western blot and in the analysis of NR2 subunits and interferon‐α and pERK change in DRG. Except in the time course study, data from western blot of the NR1 subunit, rotarod test, CFA test, and antinociceptive effect of NR1 shRNAmir in each group were analyzed by one‐way ANOVA with the Bonferroni post hoc test. Paired *t* tests were used to test the differences in NR1, NR2 subunits, and interferon‐α between the left and right sides of the DRGs. A *p*‐value of less than .05 was considered statistically significant. The analyses were performed with SPSS software (14.0; SPSS Inc., Chicago, IL, USA).

## 3 Results

### 3.1 Effects of NR1 shRNAmir1 and shRNAmir2 on formalin‐ and CFA‐induced pain

Injection of 1% formalin into the paw induced two phases of nociceptive response, with the first phase beginning immediately and persisting for 5 min. The second phase began approximately 15–20 min after injection of formalin and persisted for 20–40 min.

**Figure 1:**
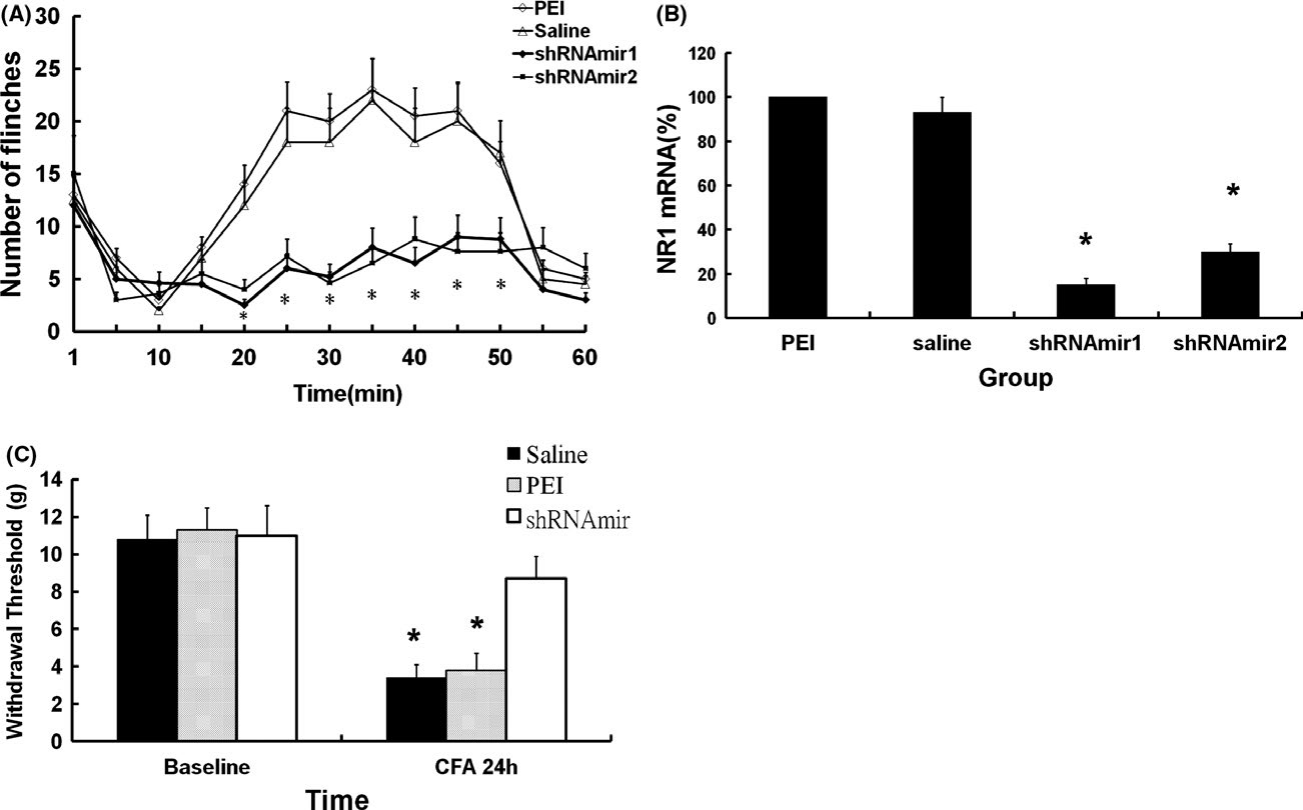
Antinociceptive and gene silencing effects of two NR1 short hairpin (sh)RNAmirs. A significant decrease in flinch number between 20 and 50 min in formalin‐induced nociceptive behavior (A) and a significant decrease in the expression of NR1 messenger RNA (mRNA) (B) were noted after injection of NR1 shRNAmir1 and shRNAmir2. *p <* *.05 NR1 shRNAmir1 and shRNAmir2 versus saline and polyethyleneimine (PEI) groups. (C) NR1 shRNAmir produced an antinociceptive effect on CFA‐induced nociception. Compared with baseline values, a significant decrease in 50% paw withdrawal threshold was noted in groups of rats that received injection of saline or PEI, but not in group of rats that received 5 μg NR1 shRNAmir1. *p <* *.05 compared with baseline values. Values are means ± *SD*s

Rats that received intradermal injections of 5 μg shRNAmir1 or shRNAmir2 showed significantly fewer formalin‐induced flinch responses during the post injection period of 20–50 min than rats that received 1 μl PEI or 100 μl saline (*p* < .05; Figure 1A). In the same rats, the NR1 mRNA levels were significantly lower than the levels in rats in the PEI and saline groups (*p* < .05) (Figure 1B). Although there was a significant reduction in NR1 mRNA level in both NR1 shRNAmir groups, the reduction was greatest in the shRNAmir1 group (85% vs. 70%; Figure 1B). Therefore, we performed the subsequent dose–response and time course studies with NR1 shRNAmir1. We further examined the antinociceptive effect of NR1 shRNAmir1 after CFA stimuli. Significant decreases in 50% PWT were noted in groups of rats that received injection of saline or PEI, but not in rats that received 5 μg NR1 shRNAmir1 (Figure 1C).

### 3.2 Dose–response antinociceptive and gene silencing effects of NR1 shRNAmir1

**Figure 2:**
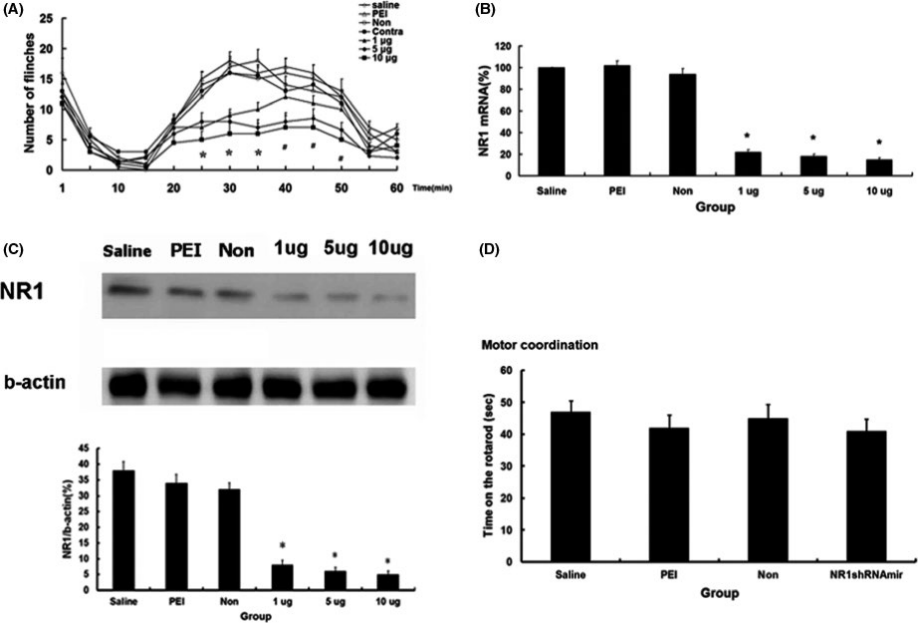
Antinociceptive effect and gene silencing effect of NR1 shRNAmir1. (A) NR1 shRNAmir1 (1, 5, and 10 μg) resulted in a significant decrease in flinch number in formalin‐induced nociceptive behavior. *p <* *.05, 1, 5, and 10 μg NR1 shRNAmir1, ^#^ *p *<* *.05, 5, and 10 μg NR1 shRNAmir1 versus saline, PEI, 10 μg nonsilencing shRNAmir, and contralateral groups. PEI, polyethyleneimine; Non, nonsilencing shRNAmir; Contra, injection of formalin on the paw contralateral to paw injected with shRNAmir. (B) NR1 shRNAmir1 (1, 5, and 10 μg) significantly inhibited the expression of NR1 messenger RNA (mRNA). (C) Administration of 1, 5, and 10 μg of NR1 shRNAmir1 significantly inhibited the expression of NR1 protein. Lanes of saline, PEI, Non: 3 days following intradermal injection of 100 μl saline, 1 μl polyethyleneimine, or 5 μg Non‐silencing shRNAmir as the control. Lanes of 1, 5, 10 μg: 3 days following intradermal injection of 1, 5, and 10 μg NR1 shRNAmir1. *p <* *.05 versus control groups (*n* = 6 each/group). (D) Motor coordination was not affected by the intradermal administration of 5 μg NR1 shRNAmir1 as measured by the rotarod test. No significant difference was noted among the four groups. Values are means ± *SD*s

Intradermal injection of 5 or 10 μg of shRNAmir1 significantly inhibited flinch responses during the period 25–50 min (*p* < .05). Flinch responses during the period 25–35 min after injection of formalin were significantly lower in rats that received intradermal injection of 1 μg of shRNAmir1 than in animals that received 1 μl PEI, 5 μg NR1 NS‐shRNAmir1, or 100 μl saline (Figure 2A; *p* < .05). There were no significant differences in the number of flinches between rats that received injection of 5 μg shRNAmir1 into the contralateral paw and those that received intradermal injection of 1 μl PEI, 5 μg NS‐shRNAmir1, or 100 μl saline (Figure 2A). Thus, the decrease in flinches induced by formalin after injection of NR1 shRNAmir1 resulted from a local rather than a systemic effect.

NR1 mRNA and protein levels were significantly lower in the 1, 5, and 10 μg NR1 shRNAmir1 groups (*p* < .05) than in the PEI, NS‐shRNAmir, and saline control groups (Figure 2B, C). There were no significant differences in mRNA or protein levels among the 1, 5, and 10 μg NR1 shRNAmir1 groups (Figure 2B,C). We selected the dose of 5 μg to study the duration of the NR1 shRNAmir1 effect on motor coordination. No significant differences were noted in time remaining on the rotarod apparatus among the rats that received 100 μl saline, 1 μl PEI, 5 μg NS‐shRNAmir1, or 5 μg NR1 shRNAmir1 (Figure 2D).

### 3.3 Time course of NR1 shRNAmir1 antinociceptive effect and gene silencing effect

**Figure 3:**
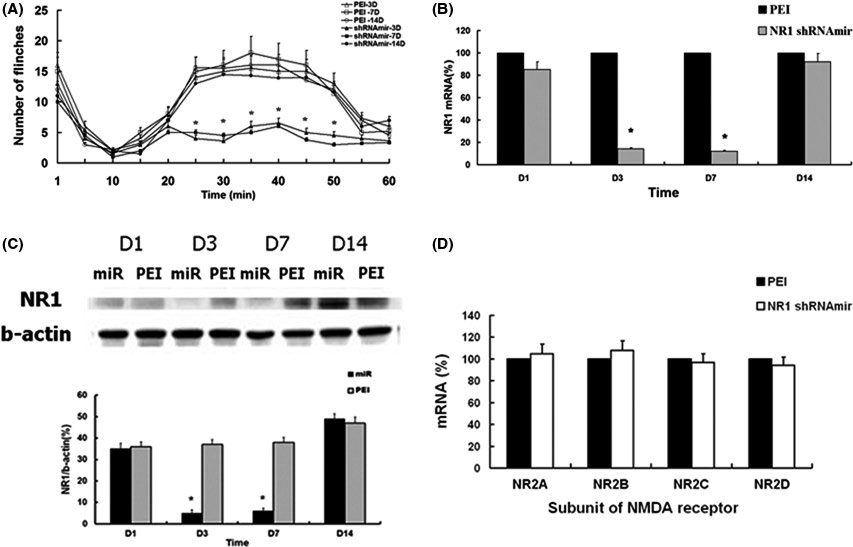
Time‐dependent antinociceptive effect and gene silencing effect of NR1 shRNAmir1. (A) Antinociceptive effect of NR1 shRNAmir1 on formalin‐induced nociceptive behavior. *p <* *.05 shRNAmir‐3D versus PEI‐3D and shRNAmir‐7D versus PEI‐7D; shRNAmir‐3D, 7D, and 14D mean tests were performed 3, 7, and 14 days after injection of 5 μg NR1 shRNAmir1; PEI‐3D, 7D, and 14D mean tests were performed 3, 7, and 14 days after injection of 1 μl polyethyleneimine. (B) Significant inhibition of NR1 messenger RNA (mRNA) was noted 3 and 7 days after injection of 5 μg NR1 shRNAmir1. (C) Significant inhibition of NR1 protein was noted at 3 and 7 days after injection of 5 μg NR1 shRNAmir1. Lanes of PEI at 1, 3, 7, and 14 days: 1, 3, 7, and 14 days following intradermal injection of 1 μl of polyethyleneimine as the control. Lanes of miR at 1, 3, 7, and 14 days: 1, 3, 7, and 14 days following intradermal injection of 5 μg NR1 shRNAmir1. Six rats were used for every time point. *p <* *.05 versus control groups (*n* = 6 each group); Values are means ± *SD*s. (D) Gene silencing effect on NR2A‐D by NR1 shRNAmir1. Intradermal injection of 5 μg NR1 shRNAmir1 did not significantly affect the expression of NR2A‐D and messenger RNA (mRNA) in the skin. mRNA of NR2A‐D subunit were measured with real‐time PCR and shown as a percentage of mRNA in the controls treated with PEI. PEI, polyethyleneimine. *p <* *.05 versus control (PEI) groups (*n* = 6 each group). Values are means ± *SD*s

Rats that received 5 μg NR1 shRNAmir1 showed significantly fewer flinch responses during the period 25–50 min after formalin pain stimulation on the third and seventh day (*p* < .05) than rats that received an intradermal injection of 1 μl PEI (Figure 3A). In addition, mRNA and protein levels of the NR1 subunit were also significantly lower on the third and seventh day (*p* < .05). On the 14th day after injection of 5 μg NR1 shRNAmir1, mRNA and protein levels of NR1 were similar to those at baseline (Figure 3B,C).

There was no significant change in the expression of NR2A‐D mRNA after injection of NR1 shRNAmir1 (Figure 3D). Furthermore, shRNAmir1 and shRNAmir2 both produced antinociceptive effects during the formalin test (Figure 1A) and inhibited the expression of NR1 (Figure 1B). Injection of NS‐shRNAmir did not affect the expression of NR1 and did not produce an antinociceptive effect (Figure 2A–C). Thus, the inhibition of NR1 expression and the antinociceptive effect produced by NR1 shRNAmir1 were specific.

### 3.4 Effect of NR1 shRNAmir1 on NR1 and pERK expression in the DRG

**Figure 4:**
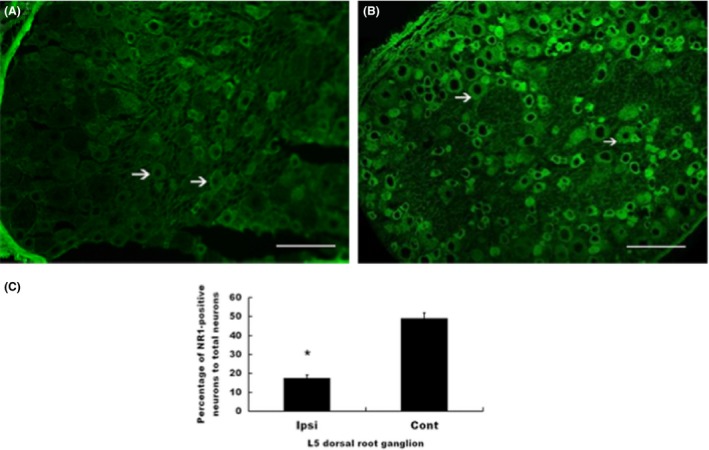
Representative immunofluorescence staining of dorsal root ganglion (DRG) 3 days after injection of 5 μg NR1 shRNAmir1. (A) Decreased fluorescence of NR1‐positive neurons indicated by arrow in DRGs ipsilateral to injection of NR1 shRNAmir1. (B) NR1 fluorescence indicated by arrow in DRGs contralateral to injection of NR1 shRNAmir1 (bar = 50 μm). (C) A significant decrease in percentage of NR1‐positive neurons was noted in DRG ipsilateral to intradermal injection of NR1 shRNAmir1. *p <* *.05 compared with contralateral DRG; ipsi, ipsilateral; cont, contralateral. Values are means ± *SD*s

**Figure 5:**
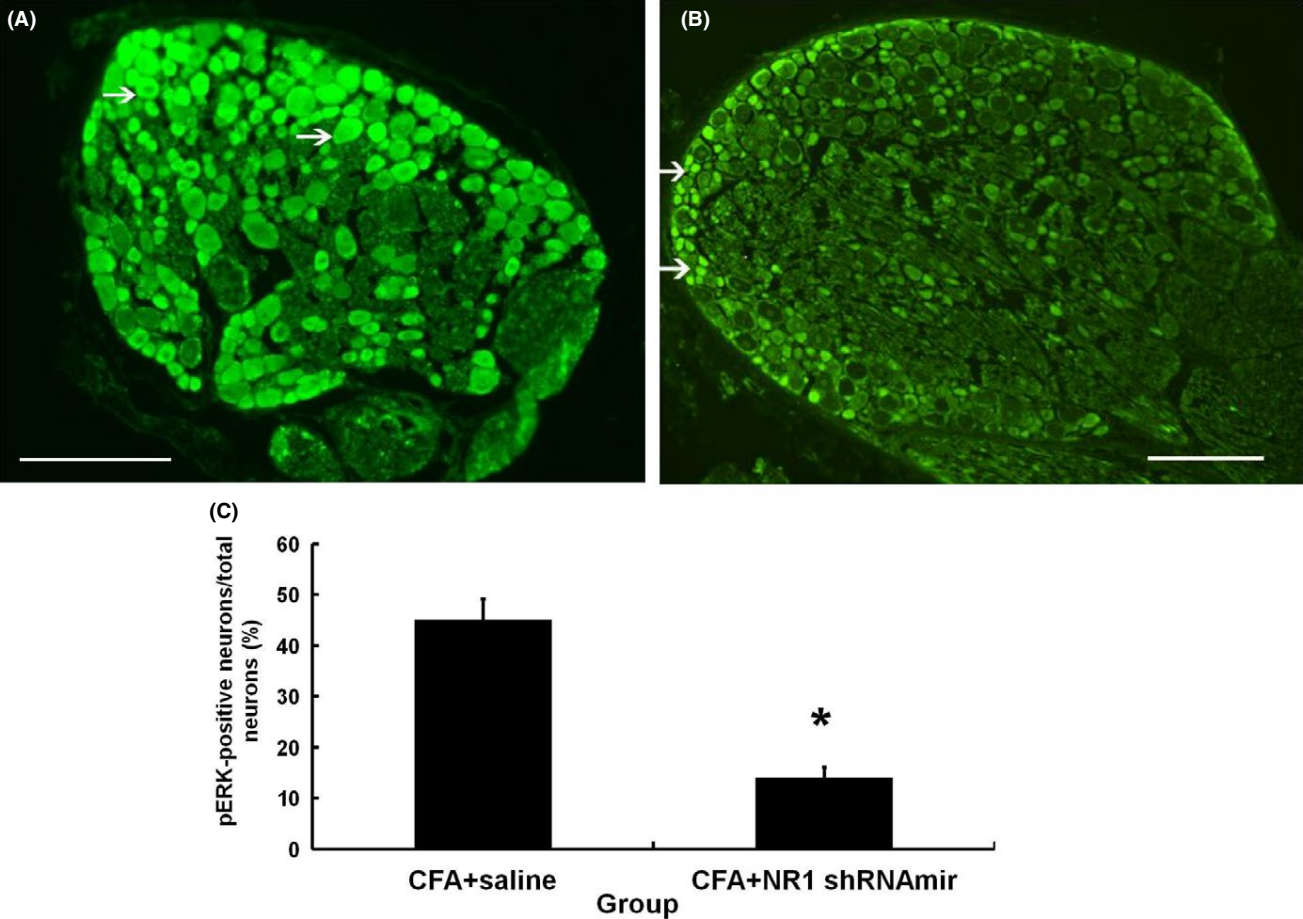
Representative immunofluorescence staining of pERK in dorsal root ganglion (DRG) 1 day after injection of CFA in rats that had received and in rats that had not received prior injection of 5 μg NR1 shRNAmir1. (A) Expression of pERK1/2 in rats that received saline only before injection of CFA. (B) Decreased expression of pERK‐positive neurons in DRGs ipsilateral to injection of NR1 shRNAmir prior to injection of CFA. (bar = 50 μm). (C) A significant decrease in percentage of pERK1/2‐positive neurons was noted in DRG ipsilateral to intradermal injection of NR1 shRNAmir1. pERK‐positive neuron is indicated by arrow. *p <* *.05 compared with DRG ipsilateral to CFA; Values are means ± *SD*s

To test the effect of local injection of NR1 shRNAmirs on the expression of NR1 in the ipsilateral DRG, we used immunofluorescence staining to measure the degree of NR1 expression in L5 DRGs. The percentage of NR1‐positive neurons in the DRG ipsilateral to the shRNAmir‐treated paw was significantly lower than that in the contralateral L5 DRG (*p* = .015; Figure 4A–C). Intense noxious stimuli have been shown to result in an NMDA receptor‐dependent increase in expression of pERK (Ji, Baba, Brenner, & Woolf, 1999). Therefore, we also examined the changes in pERK1/2 expression in DRGs ipsilateral to paws injected with CFA with or without NR1 shRNAmir. The numbers of pERK1/2‐positive neurons were significantly lower in the DRGs ipsilateral to shRNAmir‐treated paws than in the L5 DRGs ipsilateral to paws treated only with CFA (*p* = .021; Figure 5A–C). Thus, in addition to the decreased expression of NR1 in NR1 shRNAmir‐injected paw skin shown previously, the expression of NR1 in DRGs ipsilateral to NR1 shRNAmir‐injected paws was diminished by injection of NR1 shRNAmir1 in the skin. Furthermore, the expression of the pain‐related biomarker pERK under CFA stimuli was also diminished by NR1 shRNAmir treatment.

### 3.5 Effect of NR1 shRNAmir on basal nociceptive thresholds and NMDA receptor composition in DRG

**Figure 6:**
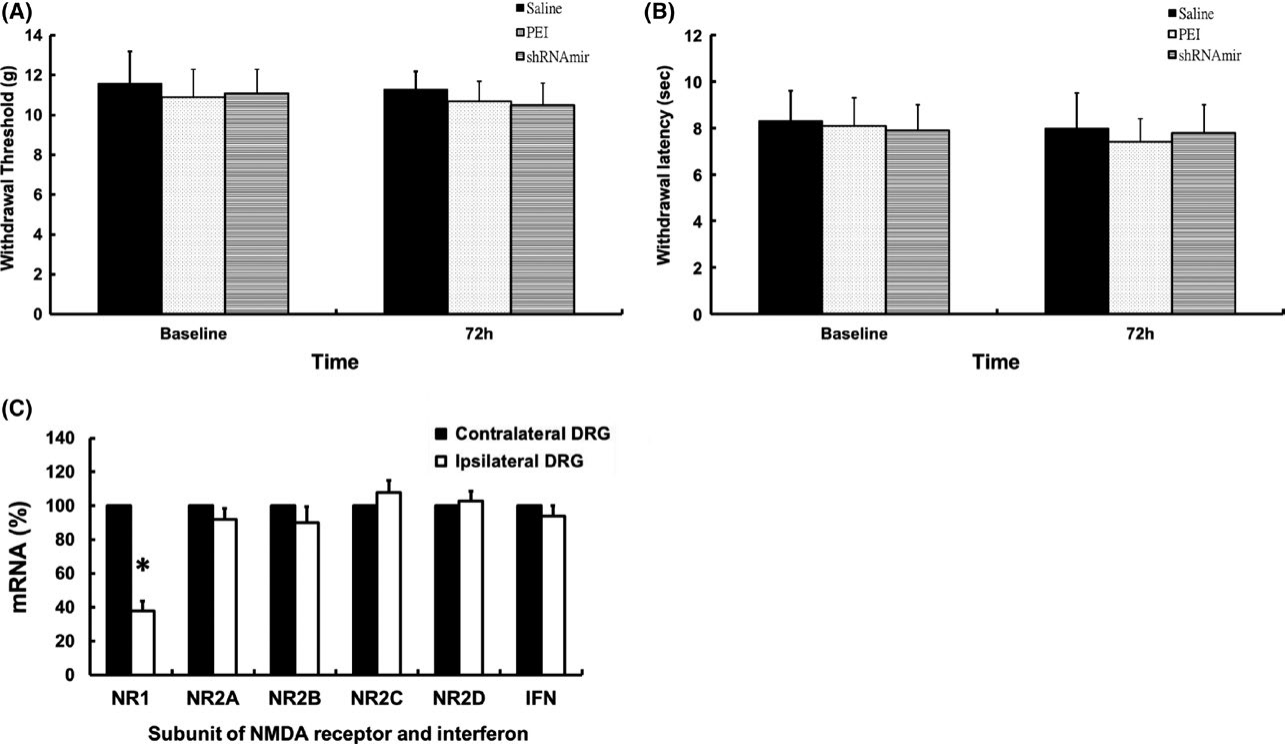
Antinociceptive effect of gene knockdown of NR1 by intradermal injection of NR1 shRNAmir and the composition of the NMDA receptor in DRG. No significant differences in mechanical withdrawal threshold (A) or thermal withdrawal latency (B) were noted after injection of 5 μg NR1 shRNAmir compared to rats that received intradermal injection of 100 μl saline or 1 μl polyethyleneimine (PEI). (C) Gene silencing effect on NR2A‐D and α‐interferon in DRG by NR1 shRNAmir1. Intradermal injection of 5 μg NR1 shRNAmir1 resulted in decreased expression of NR1, but did not significantly affect the expression of NR2A‐D or α‐interferon messenger RNA (mRNA) in the ipsilateral DRG. mRNAs of NR1, NR2A‐D subunit, and α‐interferon were measured with real‐time PCR and are shown as a percentage of mRNA in the controls of contralteral DRG. IFN: α‐interferon. *p <* *.05 versus control (contralateral DRG) groups (*n* = 4). Values are means ± *SD*s

There were no significant differences in mechanical withdrawal threshold or thermal withdrawal latency among rats 3 days after intradermal injection of 5 μg NR1 shRNAmir1, 100 μl saline, or 1 μl PEI (Figure 6A,B). There were also no significant changes in the expression levels of NR2 A‐D or interferon‐α; however, NR1 mRNA expression was significantly lower in ipsilateral DRGs than in contralateral DRGs after injection of 5 μg NR1 shRNAmir1 alone (Figure 6C). Thus, NR1 knockdown in skin and the DRG did not alter the basal nociceptive threshold or the NMDA receptor composition in the DRG. In addition, NR1 shRNAmir1 did not result in overexpression of interferon‐α, which commonly occurs in RNA interference (Figure 6C).

## 4 Discussion

*N*‐methyl‐D‐aspartate receptor inhibitors are often used for management of chronic pain because the receptor is predominantly involved in neural sensitization during the development of chronic pain. However, use of these inhibitors is limited because of the numerous side effects resulting from antagonizing NMDA receptors in the CNS (Enarson, Hays, & Woodroffe, 1999; Rabben et al., 1999; Schmid et al., 1999). Several reports have shown that NMDA receptors are present in both CNS and in the peripheral nerves (Carlton, 2001; Carlton & Coggeshall, 1999; Coggeshall & Carlton, 1998). In addition, peripheral administration of NMDA receptor antagonists can produce antinociceptive effects (Beirith et al., 2002; Davidson et al., 1997; Warncke et al., 1997). In this regard, RNA interference‐mediated gene silencing of peripheral NMDA receptors is a potential alternative for pain treatment because it provides sustained analgesic effects and avoids CNS side effects. In this study, we applied a lentiviral vector encoding a miRNA‐based shRNA that disrupts the NR1 subunit of NMDA receptors in skin and the DRG and found that the therapy provides antinociceptive effects without inducing CNS side effects.

Viral vectors are commonly used in vivo and most vectors use polymerase (pol) III promoters such as H1 and U6 or the minimal CMV promoter to drive the expression of siRNA (Xia, Mao, Paulson, & Davidson, 2002). However, excessive expression of siRNA driven by pol III promoters may result in off‐target disruption of gene expression and may induce cellular toxicity (Grimm et al., 2006; McBride et al., 2008). The gene knockdown effect of miRNA‐based shRNA whose expression is driven by pol II promoters is 12 times more potent than that of traditional shRNA whose expression is driven by pol III promoters. shRNAs have been reported to be inserted in miRNA context, for example, miR‐30‐based shRNA, which has long chain transcripts containing naturally occurring miRNA and RNAi molecules produced by the endogenous miRNA processing mechanism (Silva et al., 2005; Stegmeier, Hu, Rickles, Hannon, & Elledge, 2005; Zeng et al., 2005). Thus, we used the lentiviral miR‐30‐based shRNA vector system to drive expression of the NR1 subunit in this study. Gene silencing was evident after 3 days at a dose of 1 μg but was most pronounced at a dose of 5 μg, reflecting a fast onset of expression at very low doses of the CMV‐shRNAmir system in this study. No significant difference was found in time spent on the rotarod between rats in the shRNAmir treatment group and those in the control groups. Upon effective knockdown of the peripheral NR1 subunit, the antinociceptive effect was produced without coexisting side effects such as alterations in behavior or death.

The NR1 subunit is expressed in nearly all of the lumbar DRG neurons (Sato, Kiyama, Park, & Tohyama, 1993) and partially in the peripheral nerves (Coggeshall & Carlton, 1998). Knockdown of NR1 mRNA in the DRG may further decrease the expression of NMDA receptors on primary afferent axons, as the functional NMDA receptors are transported in and out of the cell membrane dynamically to regulate the transmission efficacy and remodel synapses (Lau & Zukin, 2007). In this study, the expression of NR1 decreased in the skin and ipsilateral DRG after subcutaneous injection of NR1 shRNAmir. Thus, the antinociceptive effect induced by local injection of shRNAmirs is hypothesized to be governed by gene silencing of the NR1 subunit in neurons of the ipsilateral DRG in addition to gene silencing of the NR1 subunit in subcutaneous sensory axons. Local injection of shRNAmir induced antinociception in the injected paw but not in the contralateral paw, which indicates that administration of shRNAmir elicits a local effect.

The formalin test is a widely used model of prolonged noxious stimulation (Dubuisson & Dennis, 1977). Several studies addressed the essential role of afferent input from the periphery in generating and maintaining nociception in the second phase of the formalin test (Dickenson & Sullivan, 1987; Pitcher & Henry, 2002). The NMDA receptor was reported to play a key role in pain hypersensitivity during the second phase of the formalin response (South et al., 2003). As shown in this study, treatment with NR1 shRNAmir inhibited the second phase response, a finding consistent with the diminished expression of the NR1 subunit after administration of NR1 shRNAmir. CFA is another inflammation‐producing chemical used to produce long‐lasting pain responses that mimic clinical pain in humans. The injection of CFA into the rat hind paw has been shown to produce mechanical hyperalgesia accompanied by long‐lasting inflammation in the injected hind paw (Stein, Millan, & Herz, 1988). NMDA receptors have been reported to play an important role in mediating the development of mechanical hyperalgesia after intraplantar injection of CFA (Leem et al., 2001). In this study, CFA‐induced mechanical hyperalgesia was attenuated by the administration of NR1 shRNAmir.

Potential adverse effects of RNAi include activation of the interferon response and off‐target gene silencing effects. Local delivery has several advantages including providing high concentration at the local site with a low dose and subsequently avoiding induction of the systemic immune response to shRNA. Interferon response was excluded in this study as interferon‐α mRNA was not significantly increased after administration of NR1 shRNAmir. In addition, off‐target effects did not occur in this study, as nonsilencing shRNAmir did not affect NR1 gene expression and NR1 shRNAmir also did not affect the expression of NR2 subunits. Thus, NR1 expression was directly inhibited by NR1 shRNAmir.

In this study, we found that miRNA‐based shRNAmir resulted in potent gene silencing and effective antinociceptive effect. One dose of 5 μg lentiviral shRNAmir produced antinociception within 3 days and the effect persisted for about 7 days. The effect of antinociception and NR1 gene silencing also appeared after injection of 1 μg NR1 shRNAmir. Thus, peripheral administration of lentiviral vector‐encoded NR1 shRNAmir offers a novel therapy for persistent inflammatory pain without affecting motor function.

## Conflicts of Interests

No competing interests declared.
